# Systematics, genetics, and biogeography of intertidal mites (Acari, Oribatida) from the Andaman Sea and Strait of Malacca

**DOI:** 10.1111/jzs.12244

**Published:** 2018-09-04

**Authors:** Tobias Pfingstl, Andrea Lienhard, Satoshi Shimano, Zulfigar Bin Yasin, Aileen Tan Shau‐Hwai, Sopark Jantarit, Booppa Petcharad

**Affiliations:** ^1^ Institute of Biology University of Graz Graz Austria; ^2^ Science Research Center Hosei University Fujimi, Chiyoda‐ku Tokyo Japan; ^3^ Centre For Marine and Coastal Studies Universiti Sains Malaysia Penang Malaysia; ^4^ School of Biological Sciences Universiti Sains Malaysia Penang Malaysia; ^5^ Excellence Center for Biodiversity of Peninsular Thailand Faculty of Science Prince of Songkla University Hat Yai Songkhla Thailand; ^6^ Department of Biotechnology Faculty of Science and Technology Thammasat University Khlong Luang District, Pathum Thani Thailand

**Keywords:** *Alismobates*, haplotype, *Indopacifica*, morphometry, Thai‐Malay peninsula

## Abstract

This study demonstrates for the first time the presence of marine‐associated mites in the Andaman Sea and Strait of Malacca and reveals a relatively high diversity of these taxa with six species from two different families: Selenoribatidae and Fortuyniidae. *Indopacifica*, a new genus of Selenoribatidae, is described from Thailand and Malaysia, with two new species, *Indopacifica pantai* n. sp. and *Indopacifica parva* n. sp. The genus is characterized by the unique combination of following characters: lacking lamellar ridges, incomplete dorsosejugal suture, fourteen pairs of notogastral setae, and presence of epimeral foveae. A phylogenetic reconstruction based on *18S* ribosomal RNA sequences clearly confirms the distinctness of the new genus *Indopacifica* and places it close to the genus *Rhizophobates*. The lack of molecular genetic data of possible relatives impedes a clear assessment, and hence, we emphasize the need for further combined approaches using morphological and molecular genetic sequence data. All species show wide distribution areas within this geographic region suggesting that these taxa are good dispersers despite their minute size and wingless body. Molecular genetic data demonstrate recent gene flow between far distant populations of *I. pantai* n. sp. from the coasts of Thailand and two islands of Malaysia and hence confirm this assumption. The seasonally changing surface currents within this geographic area may favor hydrochorous dispersal and hence genetic exchange. Nevertheless, morphometric data show a slight trend to morphological divergence among the studied populations, whereas this variation is suggested to be a result of genetic drift but also of habitat differences in one population of *Alismobates pseudoreticulatus*.

## 1 INTRODUCTION

The Andaman Sea is an active back‐arc basin where the Indian and the Burma plate collide, and therefore, it is an area of increased seismic activities (Radhakrishna, Lasitha, & Mukhopadhyay, 2008). This area was also strongly affected by the marine earthquake of the December 26, 2004, which caused a tragic tsunami event costing thousands of lives and devastating coastal environments of the whole Indo‐Pacific area.

Despite this catastrophic impact which also resulted in a measurable loss of biodiversity (e.g. Adger, Hughes, Folke, Carpenter, & Rockstrom, 2005; Kumaraguru, Jayakumar, Jerald Wilson, & Ramakritina, 2005), the Andaman Sea and adjacent regions are one of the most biodiversity‐rich regions and are important biodiversity hot spots (Myers, Mittermeier, Mittermeier, da Fonseca, & Kent, 2000). Moreover, the true inventory of coastal and marine biodiversity could be several times higher than what is known today as only reports of commercially important groups, for example, fishes or molluscs are very detailed but they are scarce with respect to the minor phyla (Venkataraman & Wafar, 2005).

Indeed, intertidal oribatid mites are one of these minor groups and the reports from this geographic area remain poorly known, only records of three species from the nearby region of Singapore were recently published, namely *Alismobates pseudoreticulatus* Pfingstl, 2015, *Fortuynia smiti* Ermilov, Tolstikov, Mary, & Schatz, 2013 and *Selenoribates asmodeus* Pfingstl, 2015 (Pfingstl, 2015a,b; Pfingstl & Schuster, 2014). These tiny animals belong to the superfamily of Ameronothroidea, containing the Ameronothridae, Podacaridae, Fortuyniidae, and Selenoribatidae. The Ameronothridae and Podacaridae are exclusively occurring in cold‐temperate and polar areas, and the Fortuyniidae and Selenoribatidae, on the other hand, are restricted to the subtropical and tropical zones (e.g. Pfingstl, 2017). They are air‐breathing arthropods having evolved a littoral lifestyle, now leading a life between the tides (e.g. Pfingstl, 2017). They use elaborate plastron respiration systems to withstand flooded conditions during high tide (e.g. Pfingstl & Krisper, 2014; Pugh, King, & Fordy, 1990), feed on intertidal algae, and occupy a wide range of coastal habitats, as, for example, rocky cliffs, boulder beaches, or mangrove forests (Pfingstl, 2013a). Some species are known to be island endemics, whereas others are distributed across large archipelagos and oceanic regions (Pfingstl & Schuster, 2014). In the latter case, populations from different islands or regions may show morphological diversification that is related to geographic distance between the landmasses and these divergences are supposed to indicate ongoing speciation processes due to restricted gene flow between populations (Pfingstl & Baumann, 2017; Pfingstl & Jagersbacher‐Baumann, 2016). How these tiny flightless arthropods disperse between the islands is still unknown but several authors argue that drifting along ocean currents is the most likely mode of long distance transport (Coulson, Hodkinson, Webb, & Harrison, 2002; Pfingstl, 2017; Schatz, 1991). Another interesting evolutionary phenomenon, namely cryptic diversity, was also demonstrated in these intertidal mites, with species occurring on the same island, possessing almost identical appearance but occupying different ecological niches within the intertidal habitat (Pfingstl, Lienhard, & Jagersbacher‐Baumann, 2014).

The number of described species of the subtropical and tropical Fortuyniidae and Selenoribatidae nearly doubled in the last decade. Presently, the Fortuyniidae contain four genera (*Alismobates*,* Circellobates*,* Fortuynia,* and *Litoribates*) with 26 species, whereas the Selenoribatidae comprise eight genera (*Arotrobates*,* Carinozetes*,* Psednobates*,* Rhizophobates*,* Schusteria*,* Selenoribates*,* Thalassozetes,* and *Thasecazetes*). Despite these recent findings, knowledge about the evolution, phylogeny, and distribution of these taxa is still largely incomplete and even existing systematic classifications remain controversial. For example, the generic diagnosis of the selenoribatid *Schusteria* Grandjean, 1968 has repeatedly been subject to misinterpretations and erroneous taxonomic actions (Pfingstl & Schuster, 2012) leading to a blurry picture of this taxon and closely related genera. A recent molecular genetic study even questioned the monophyletic status of the family Fortuyniidae suggesting that certain fortuyniid taxa may indeed belong to the Selenoribatidae (Iseki & Karasawa, 2014). Apart from these systematic problems, the diversity of both families is largely underestimated as indicated by the recent discovery of numerous new species and genera from the Red Sea, the Eastern Pacific, the Indo‐Pacific, and the Caribbean area (e.g. Pfingstl, 2015a; Pfingstl, Baumann, Lienhard, & Schatz, 2017; Pfingstl & Jagersbacher‐Baumann, 2016; Pfingstl & Schatz, 2017).

During an international expedition to investigate the biodiversity of interstitial and intertidal habitats of selected coastal areas of Malaysia and Thailand, littoral mites were found at various sampling sites. This material contained two new selenoribatid taxa but also three fortuyniid species known from far distant areas. Presently, nothing is known about the dispersal abilities and gene flow between the populations of these intertidal mite species showing such wide distribution areas with enormous oceanic barriers in between. Therefore, the aims of this study were (a) to document and discuss the biogeographic pattern found for oribatid mites in the Andaman Sea; (b) to compare distant populations of supposedly widespread species with morphometric and molecular genetic approaches in order to assess dispersal abilities and gene flow; (c) to describe a new genus with two species; and (d) to provide the first insight into the biodiversity of intertidal mites from this biologically interesting geographic region.

## 2 MATERIALS AND METHODS

Samples of intertidal algae were scraped off rocks with a knife during low tide. Algae were then put in Berlese‐Tullgren funnels for approx. 24 hr to extract mites. Afterward, mites were removed from the collecting vessel by hand with a brush and stored in absolute (100%) ethanol for morphological and molecular genetic investigation.

Morphological terminology used in this study follows that of Grandjean, 1953 and Norton & Behan‐Pelletier, 2009. Formulas for leg setation are provided in parentheses according to the sequence trochanter–femur–genu–tibia–tarsus followed by formulas for leg solenidia also given in parentheses according to the sequence genu–tibia–tarsus.

### 2.1 Sample locations

The Andaman Sea is situated in the eastern part of the Indian Ocean and lies southeast of the Bay of Bengal for overview map see Figure S1. It extends from the Andaman and Nicobar Islands in the West to the coast of southern Myanmar in the North downward to the Thai‐Malay peninsula and to the Strait of Malacca in the South. The latter is a narrow body of water between the Malay Peninsula and the Indonesian island of Sumatra (for an overview map of the region please see Supporting Information Figure S1).

The climate of the Andaman region is strongly influenced by the tropical monsoons of Southeast Asia with wind systems reversing directions every year. Consequently, the climate is greatly influencing the hydrographic parameters and water movement in the area (e.g. Kiran, 2017; Wyrtki, 1961).

Sampling locations were as follows: Langkawi, Malaysia: (a) Pantai Legenda; *Bostrychia* sp. (red intertidal algae) growing on boulder, upper eulittoral (MY_05), coordinates 6°18′42.91′′N 99°51′06.47′′E; October 24, 2016. (b) Pantai Pasir Hitam; diverse algae growing on rock, upper eulittoral (MY_07), coordinates 6°25′24.24′′N 99°47′15.81′′E; October 25, 2016. (c) Datai Bay; *Bostrychia* sp. growing on rock, upper eulittoral (MY_11), coordinates 6°26′01.39′′N 99°41′03.17′′E; and red filiform algae on stones, lower eulittoral (MY_12), coordinates 6°26′02.04′′N 99°40′54.51′′E; October 26, 2016.

Penang, Malaysia: (d) Pantai Pasir Panjang; *Bostrychia* sp. growing on large rocks, upper eulittoral (MY_17), coordinates 5°18′01.61′′N 100°11′03.95′′E; October 28, 2016.

Phang Nga province, Thailand: (e) Nang Thong Beach, Takua Pa district; short brown algae covering boulder; medium eulittoral (TH_06), coordinates 8°37′46.76′′N 98°14′35.95′′E; November 6, 2016. (f) Nang Thong Beach; *Bostrychia* sp. growing in crevice, upper eulittoral (TH_09), coordinates 8°38′07.63′′N 98°14′39.77′′E; November 8, 2016.

### 2.2 Genetic analyses

Seventy‐nine specimens of all ameronothroid mites (see Appendix) collected in Thailand and Malaysia were analyzed. Therefore, total genomic DNA was extracted from single individuals preserved in absolute ethanol. Extraction was carried out using the Chelex method (Casquet, Thebaud, & Gillespie, 2012) with the following adjustments: Whole specimens were crushed against the tube wall in microcentrifuge tubes containing 55 μl of a 10% Chelex solution (with 2 μl Proteinase K). Samples were extracted for 3–4 hr at 56°C. Three gene fragments were sequenced for this study: the mitochondrial *cytochrome c oxidase subunit 1* gene (*COI*), the nuclear *elongation factor 1 alpha* gene (*EF‐1*α), and the nuclear *18S* rRNA gene (*18S*). A 567‐bp fragment of the *COI* gene was amplified using the primer pairs Mite COI‐2F and Mite COI‐2R (Otto & Wilson, 2001), and for the 513 bp containing *EF‐1*α gene fragment, the primer 40.71F and 52.RC (Regier & Shultz, 1997) were used. The complete *18S* rRNA gene (~1.8 kb) was amplified in two overlapping fragments according to the PCR protocol of Dabert, Witalinski, Kazmierski, Olszanowski, and Dabert (2010) using the recommended primers (Skoracka & Dabert, 2010). Primer sequences are given in Supporting Information Table S4. PCR conditions for the *COI* gene fragment are given in Pfingstl et al. (2014) and those for the *EF‐1*α gene fragment in Lienhard, Schäffer, Krisper, and Sturmbauer (2014). DNA purification (with the enzyme cleaner ExoSAP‐IT, Affymetrix; and the Sephadex G‐50 resin, GE Healthcare) and sequencing steps (using the BigDye Sequence Terminator v3.1 Cycle Sequencing Kit, Applied Biosystems) were conducted after the methods published by Schäffer, Krisper, Pfingstl, and Sturmbauer (2008). Sequencing was performed in both directions on an automated capillary sequencer (ABI PRISM 3130xl, Applied Biosystems).

Alignments were generated by means of the program MEGA6 (Tamura, Stecher, Peterson, Filipski, & Kumar, 2013). For all gene fragments, Bayesian 50% majority rule consensus trees were generated by means of MrBAYES 3.1.2 (Ronquist & Huelsenbeck, 2003) applying a MC^3^ simulation with 20 million generations (five chains, two independent runs, 10% burn‐in, GTR + I + G model). Results were analyzed in TRACER v.1.6 (Rambaut & Drummond, 2007) to check for convergence and to ensure the stationarity of all parameters. Neighbor joining (NJ) trees were generated with MEGA6 (5000 bootstrap replicates) and maximum‐likelihood (ML) analyses were carried out using RAxML (Stamatakis, 2014) applying 5000 bootstrap replicates and the GTR + gamma model.

To determine the geographic correspondence with the genetic structure, TCS networks were constructed with the program PopART (Leigh & Bryant, 2015, http://popart.otago.ac.nz) applying default settings. Uncorrected *p*‐distances were calculated in MEGA6.

All sequences obtained from this study were deposited in GenBank (www.ncbi.nlm.nih.gov/genbank; accession numbers for *COI*: MH285595–MH285673, *EF‐1*α: MH285674–MH285689, and *18S*: MH285690–MH285696; more details are given in the Appendix). For the *18S* dataset, all already published ameronothroid sequences were also integrated into the alignment.

### 2.3 Morphometric analyses

**Figure 1:**
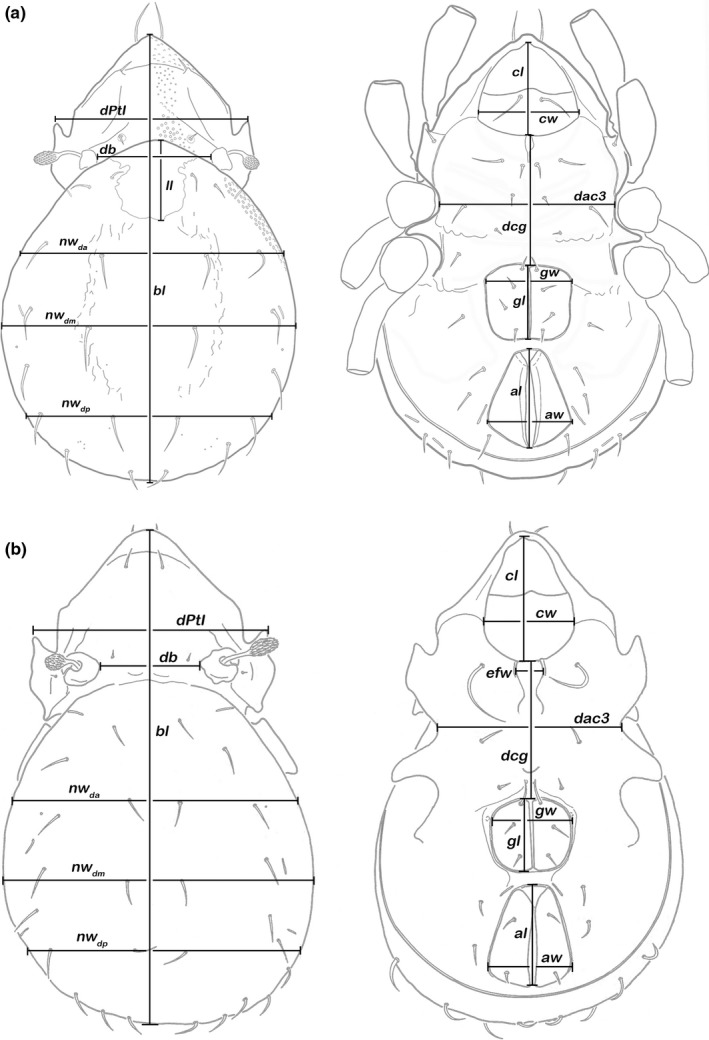
Graphic illustration of measured continuous variables. (a) *Alismobates pseudoreticulatus*. (b) *Indopacifica pantai* n. sp. Dorsal aspect: *bl*: body length; *dPtI*: distance between pedotecta I, *db*: distance between bothridia; *ll*: lenticulus length; *nw*_*c1*_: notogastral width on level of seta *c*_*1*_; *nw*_*da*_: notogastral width on level of seta *da*;* nw*_*dm*_: notogastral width on level of seta *dm*. Ventral aspect: *cl*: camerostome length; *cw*: camerostome width; *efw*: epimeral fovea width; *dcg*: distance between camerostome and genital orifice; *dac3*: distance between acetabula 3; *gl*: genital orifice length; *gw*: genital orifice width; *al*: anal opening length; *aw*: anal opening width

For morphometric investigation, 81 specimens in total (not the same as used in molecular genetic analyses) were placed in lactic acid (temporary slides) and measurements were performed using a compound light microscope (Olympus BH‐2) and ocular micrometre. A total of 15 continuous variables were measured in 30 specimens of *A. pseudoreticulatus* from three different localities on Langkawi (MY_05, MY_07, MY_11), and 15 continuous variables were taken in 51 specimens of *Indopacifica pantai* n. sp. from the island of Penang (MY_17) in Malaysia and from Phang Nga province (TH_09) in Thailand (Figures 1a,b).

For univariate statistics minimum, maximum, mean, standard deviation, and coefficient of variation (cv) were calculated. These numbers were calculated to assess variation within but also between populations. Mann–Whitney *U* test was used for comparing the means of variables for pairwise comparisons in order to clarify if single variables differ significantly between two populations.

Principal component analysis (PCA) was performed on log_10_‐transformed raw and size‐corrected data using a variance–covariance matrix. No rotation was applied to the multivariate data. Size correction was done by dividing each variable through the geometric mean of the respective specimen (e.g. Jagersbacher‐Baumann, 2014; Pfingstl & Jagersbacher‐Baumann, 2016). All analyses were performed with PAST 3.11 (Hammer, Harper, & Ryan, 2001).

### 2.4 Drawings and photographs

For microscopic investigation in transmitted light, preserved animals were embedded in Berlese mountant. Drawings were made with an Olympus BH‐2 Microscope equipped with a drawing attachment. These drawings were first scanned, then processed, and digitized with the free and open‐source vector graphics editor Inkscape (https://inkscape.org).

For photographic documentation, specimens were air‐dried and photographed with a Keyence VHX‐5000 digital microscope.

## 3 RESULTS

### 3.1 Molecular genetics

**Figure 2:**
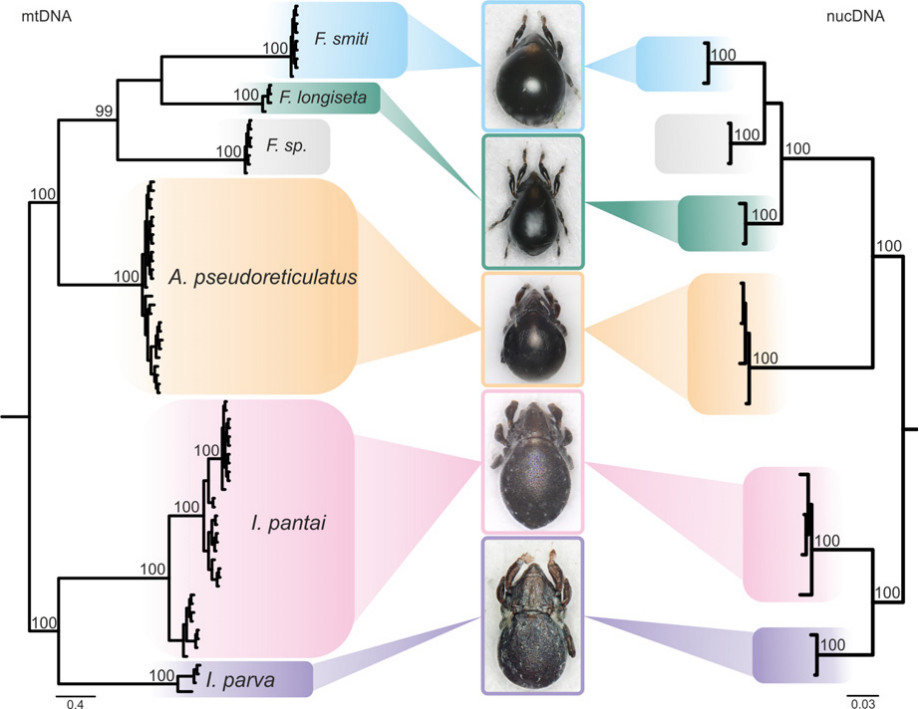
Bayesian inference trees based on *COI* (left) and *EF‐1*α (right) sequences. Species are grouped in colored boxes with corresponding micrographs of adult individuals. Posterior probabilities (>95%) are shown above branches

**Figure 3:**
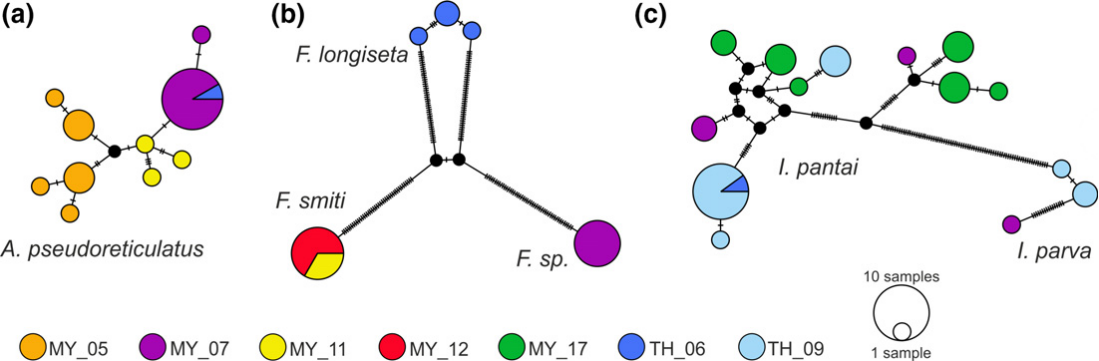
TCS haplotype networks based on *COI* sequences including three ameronothroid genera, namely *Alismobates* (a), *Fortuynia* (b), and *Indopacifica* (c). Each circle corresponds to one haplotype, and its size is proportional to its frequency, the number of mutations are indicated as hatch marks. Small black circles represent intermediate haplotypes not present in the dataset. Colors refer to different locations in Malaysia and Thailand and correspond to those in Figure 11a,b

**Figure 4:**
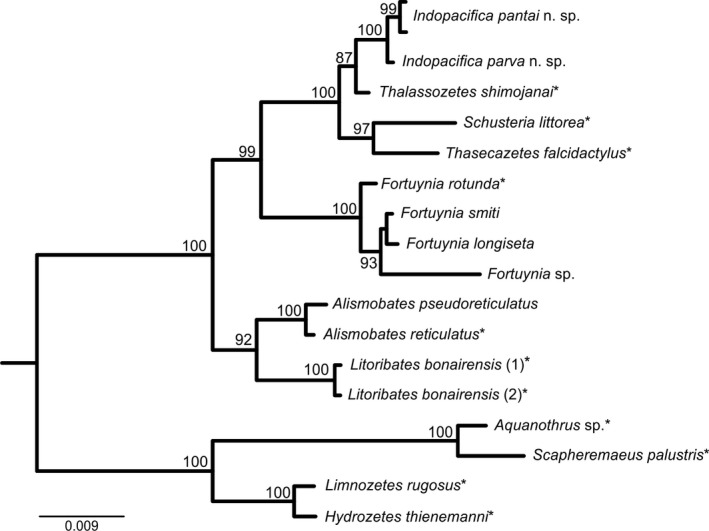
Bayesian inference tree based on *18S* sequences. Posterior probabilities (>80) are shown on the nodes. Sequences obtained from GenBank are marked by an asterisk (*)

Bayesian inference gene trees, based on the mitochondrial (*COI*) as well as on the nuclear marker (*EF‐1*α), revealed six highly divergent, well supported (all posterior probability values 100) and monophyletic lineages referring to six distinct species (Figure 2). Because of the higher statistical support, BI topologies are shown herein, although NJ and ML analyses resulted in the exact same clades. Within the Selenoribatidae, two clades are present harboring two new species of the genus *Indopacifica* n. gen., and within the Fortuyniidae, one lineage refers to *A. pseudoreticulatus* and three further clades represent the genus *Fortuynia* (*F. smiti*,* F*. sp., and *F. longiseta*). The distinctness of the species is demonstrated by the clear gap between mean intra‐ and interspecific *p*‐distances of both markers (3.0 vs. 14.6% *COI*; 0.9 vs. 6.5% *EF‐1*α; Table 1). For the *COI* gene fragment, the highest intraspecific *p*‐distance amounted to 6.4% (*I. pantai* n. sp.) compared to the lowest interspecific distance of 14.0%. For the *EF‐1*α gene fragment, the highest intraspecific *p*‐distance reached 1.6% (*I. pantai* n. sp.) compared to the lowest interspecific distance of 6.3% between two fortuyniid species. *Alismobates pseudoreticulatus* as well as both *Indopacifica* species are distributed in Malaysia and Thailand, but haplotypes of *A*. *pseudoreticulatus* are clearly associated with their geographic distribution (Figures 3a,b and 11a,b). *Indopacifica pantai* n. sp. as well as *I. parva* n. sp., on the other hand, show no clear geographic structure (Figures 3a,b and 11a). Data for fortuyniid species were less comprehensive; therefore, only one haplotype for *Fortuynia* sp. and *F. smiti* and three different haplotypes for *F. longiseta* were detected. To resolve deeper splits in the phylogeny and to compare the phylogenetic position of the new genus and species to already published data, *18S* sequences were gained and compared (Figure 4). These sequences correspond to known ones, and no conflicting positions were detected. The monophyly of the new genus *Indopacifica* is strongly supported, and the two species are clearly distinct from each other.

**Table 1:** Intra‐ and interspecific mean *p*‐distances given for the *COI* (lower‐left) and the *EF‐1*α (upper‐right) gene fragment in percent

	*Fortuynia smiti* (2)	*Fortuynia longiseta* (2)	*Fortuynia* sp. (2)	*Alismobates pseudoreticulatus* (4)	*Indopacifica pantai* (4)	*Indopacifica parva* (2)
*Fortuynia smiti* (9)	0.0/0.4	8.4	7.1	15.0	14.8	14.5
*Fortuynia longiseta* (4)	15.4	0.4/0.4	6.5	15.4	14.9	14.9
*Fortuynia* sp. (7)	15.9	15.9	0.0/0.0	15.8	15.6	14.9
*Alismobates pseudoreticulatus* (25)	15.5	16.1	14.6	0.5/0.3	14.8	13.8
*Indopacifica pantai* (30)	17.5	16.1	18.7	15.5	3.0/0.9	9.1
*Indopacifica parva* (4)	16.0	18.7	16.8	14.6	17.0	1.6/0.4

*Note*

The number of investigated specimens is given in parenthesis.

### 3.2 Morphometry

#### 3.2.1 Univariate statistics of *Alismobates pseudoreticulatus* populations

The *A. pseudoreticulatus* populations from Langkawi differed highly significantly (*p* < 0.001) in body length (*bl*) and posterior notogastral width (*nw*_*dp*_) when compared by Mann–Whitney *U* test (Table 2). The specimens from the north shore (MY_07) were slightly longer and broader than the specimens from the south shore (MY_05). The variability as indicated by the coefficient of variation (cv) was moderate in all populations with values hardly exceeding 0.05. The most variable characters were the lenticulus length (*ll*) and the posterior notogastral width (*nw*_*dp*_).

**Table 2:** Univariate statistics for *Alismobates pseudoreticulatus* populations from three different locations (MY_05, MY_07, and MY_11) on the island of Langkawi

Variables	*Alismobates pseudoreticulatus*				
	MY_05 (*N* = 15)	MY_07 (*N* = 14)	MY_11 (*N* = 1)	cv	MWU
*bl*	295–325 (309 ± 7.69)	306–332 (319 ± 6.76)	302	0.03	*
*dPtI*	135–147 (143 ± 2.89)	140–148 (143 ± 2.39)	142	0.02	**
*db*	77–86 (91 ± 3.05)	77–86 (82 ± 2.10)	83	0.03	**
*ll*	55–71 (64 ± 4.52)	55–71 (62 ± 5.20)	55	**0.08**	**
*nw*_*da*_	197–231 (211 ± 9.09)	206–231 (216 ± 7.64)	206	0.04	**
*nw*_*dm*_	209–234 (221 ± 6.87)	219–240 (229 ± 5.55)	225	0.03	**
*nw*_*dp*_	169–200 (180 ± 7.76)	179–212 (193 ± 8.77)	200	**0.06**	*
*cl*	77–86 (81 ± 3.18)	77–86 (80 ± 2.59)	80	0.04	**
*cw*	68–80 (74 ± 3.18)	69–77 (73 ± 1.91)	71	0.04	**
*dcg*	68–80 (73 ± 3.25)	71–80 (76 ± 3.32	74	0.05	**
*dac3*	123–132 (127 ± 2.66)	123–132 (128 ± 2.27)	129	0.02	**
*gl*	43–52 (49 ± 2.36)	46–55 (49 ± 2.82)	46	0.05	**
*gw*	55–65 (59 ± 2.53)	59–66 (61 ± 2.01)	59	0.04	**
*al*	65–71 (67 ± 2.07)	65–75 (70 ± 2.42)	68	0.04	**
*aw*	52–62 (57 ± 2.61)	55–62 (58 ± 2.16)	59	0.04	**

*Notes.* Minimum–maximum (mean ± standard deviation) of each measured variable given in μm; cv—coefficient of variation, values higher than 0.5 are given in bold.

MWU—Mann–Whitney *U* test (comparing the medians of the populations). Abbreviations for variables are explained in caption of Figure 1.

*p < 0.001, **p > 0.001; single specimen from MY_11 not included in this test.

#### 3.2.2 Multivariate analysis of *Alismobates pseudoreticulatus*

**Figure 5:**
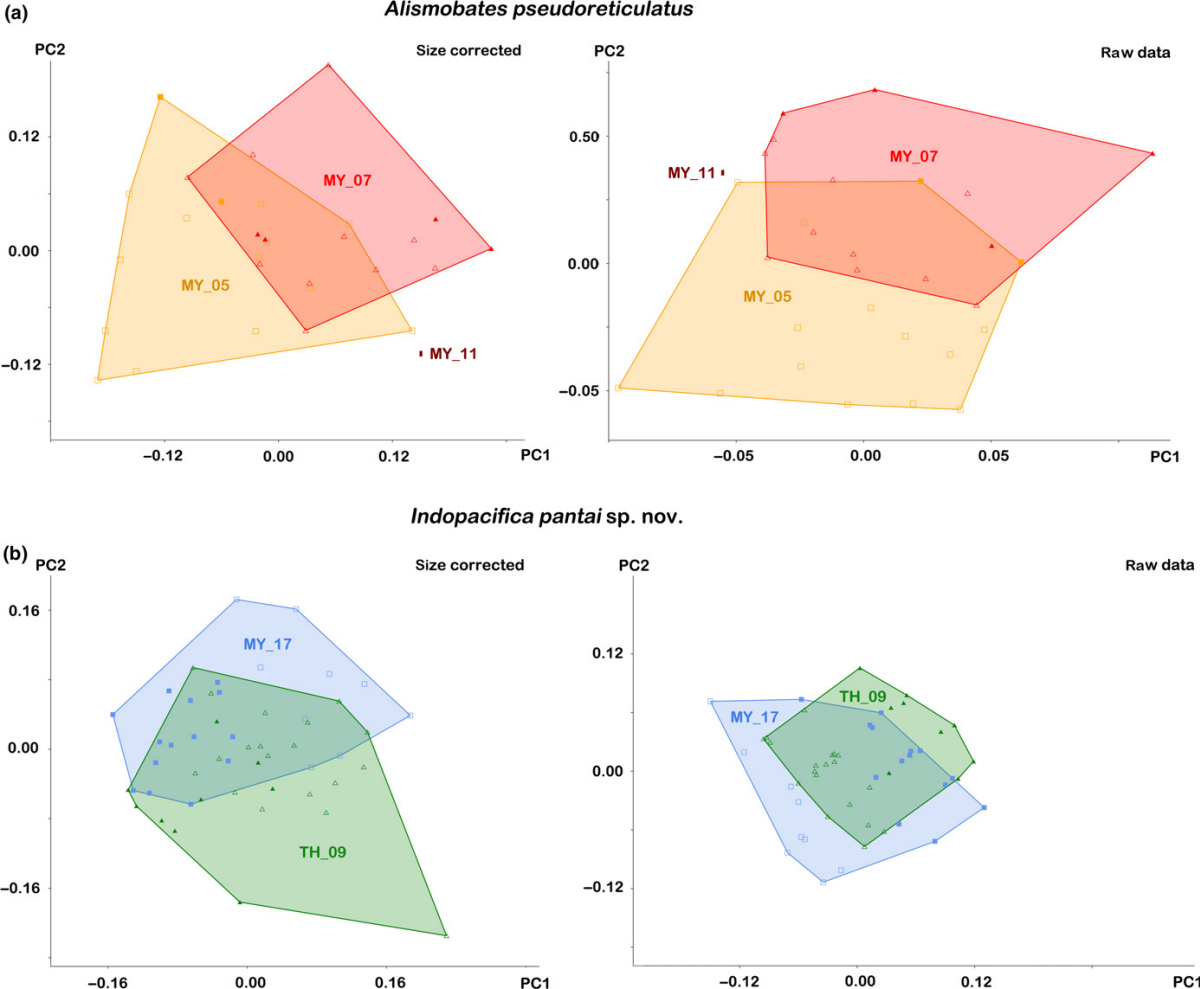
Graphs showing results of principal component analyses performed with raw data and size‐corrected data of two different species from different locations in the Andaman Sea and the Strait of Malacca. (a) *Alismobates pseudoreticulatus*. (b) *Indopacifica pantai* n. sp. Different populations represented by different colors. Open symbols refer to males, and filled symbols refer to female specimens. Codes (e.g. MY_05) refer to sample locations

Principal component analysis on both raw and size‐corrected data showed a slight misalignment of the populations indicating a trend toward morphological divergence (Figure 5a). In raw data, PC1 accounted for 37.14%, PC 2 for 27.04%, and PC3 for 9.82% of total variation and loadings higher than 0.5 are given for the lenticulus length (*ll*) and for the posterior notogastral width (*nw*_*dp*_) (Supporting Information Table S1). Similar results were shown for the size‐corrected data; PC1 is responsible for 49.51%, PC2 for 22.53%, and PC3 for 7.39% of total variation, whereas the variables *bl* and *nw*_*dp*_ showed loadings with remarkably high values (>0.5).

#### 3.2.3 Univariate statistics of *Indopacifica pantai* n. sp. populations

*Indopacifica pantai* n. sp. populations from Malaysia and Thailand only differed significantly (*p* < 0.001) in the distance between bothridia (*db*) as indicated by Mann–Whitney *U* test (Table 3). The bothridia of the Thai specimens (TH_09) were slightly farther apart than in the Malaysian specimens (MY_17). The variability was basically low, only the epimeral fovea (*efw*) and the size of genital orifice (*gl*,* gw*) show higher coefficients of variation with 0.13 and 0.09, respectively. Except *efw*, these variables are related to a moderate sexual dimorphism.

**Table 3:** Univariate statistics for *Indopacifica pantai* n. sp. populations from two different locations in the Andaman Sea and the Strait of Malacca

Variables	*Indopacifica pantai*			
	MY_17/Penang (*N* = 23)	TH_09/Phang Nga (*N* = 28)	cv	MWU
*bl*	319–356 (333 ± 9.84)	319–350 (332 ± 8.10)	0.03	**
*dPtI*	139–154 (147 ± 3.88)	142–154 (148 ± 2.71)	0.02	**
*db*	52–68 (63 ± 3.44)	61–71 (66 ± 2.17)	0.05	*
*nw*_*da*_	175–206 (192 ± 7.94)	185–206 (194 ± 5.89)	0.04	**
*nw*_*dm*_	191–215 (205 ± 5.98)	197–219 (208 ± 5.69)	0.03	**
*nw*_*dp*_	163–185 (175 ± 6.41)	169–194 (181 ± 6.33)	0.04	**
*cl*	83–92 (90 ± 2.42)	62–94 (90 ± 5.68)	0.05	**
*cw*	62–68 (65 ± 1.79)	62–68 (65 ± 1.50)	0.02	**
*efw*	19–34 (28 ± 3.74)	22–31 (26 ± 2.93)	**0.13**	**
*dcg*	71–83 (76 ± 3.75)	74–86 (80 ± 3.35)	0.05	**
*dac3*	111–123 (116 ± 3.23)	108–120 (117 ± 2.64)	0.03	**
*gl*	40–55 (48 ± 4.72)	42–55 (48 ± 3.91)	**0.09**	**
*gw*	46–59 (53 ± 5.17)	49–62 (54 ± 4.86)	**0.09**	**
*al*	71–77 (74 ± 2.08)	69–77 (73 ± 1.93)	0.03	**
*aw*	52–59 (55 ± 2.62)	52–62 (57 ± 2.53)	0.05	**

*Notes.* Minimum–maximum (mean ± standard deviation) of each measured variable given in μm; cv—coefficient of variation, values higher than 0.5 are given in bold.

MWU—Mann–Whitney *U* test (comparing the medians of the two populations). Abbreviations for variables are explained in caption of Figure 1.

*p < 0.001, **p > 0.001.

#### 3.2.4 Multivariate analysis of *I. pantai* n. sp

The PCA on raw and size‐corrected data resulted in mainly overlapping clusters between the populations from Malaysia and Thailand with a slight displacement on PC2 axis (Figure 5b). In the raw data, PC1 was responsible for 43.57%, PC2 for 26.29%, and PC3 for 9.85% of total variation. Variables with high loadings (>0.5) were the epimeral fovea (*efw*) and the genital orifice (*gw*), the latter is subject to a moderate sexual dimorphism (Supporting Information Table S2). Using size‐corrected data, PC1 accounted for 28.77% of total variation, PC2 for 18.5%, and PC3 for 14.66%. Loadings with high values were found for the body length (*bl*) and the posterior notogastral width (*nw*_*dp*_). Individuals from Thailand tended to be slightly shorter but the posterior body region was slightly larger than in specimens from Malaysia.

### 3.3 Records of known species

3.3.1

**Fortuyniidae Hammen, 1963**

***Alismobates* Luxton, 1992**

***Alismobates pseudoreticulatus* Pfingstl, 2015 (Supporting Information Figure** S2a)

###### Present records

Malaysia, Langkawi (Legenda, Pantai Hitam Pasir, Datai Bay) and Thailand, Phang Nga province (Nang Thong Beach).

###### Biogeographic and morphological remarks

This species was originally reported from the coasts of Singapore (Pfingstl, 2015b). The specimens from Malaysia and Thailand (body size 295–322 μm) do not differ morphologically from the type specimens from Singapore (body size 292–308 μm). Although not mentioned in the original description (Pfingstl, 2015b), specimens from all populations possess an obvious median sternal globular projection adjacent to the border of the camerostome.

***Fortuynia* Hammen, 1960**

***Fortuynia smiti* Ermilov et al.,** 2013 **(Supporting Information Figure** S2b)

###### Present records

Malaysia, Langkawi (Pantai Hitam Pasir, Datai Bay) and Thailand, Phang Nga province (Nang Thong Beach).

###### Biogeographic and morphological remarks

This species was first discovered in New Caledonia (Ermilov et al., 2013), and later it was also found on the coasts of Singapore (Pfingstl, 2015b). The present specimens do not show any morphological difference to the above‐mentioned individuals. Their body size (563–625 μm) is also well in accordance with the size range of the New Caledonian individuals (564–614 μm) and the specimens from Singapore (552–589 μm).

##### *Fortuynia longiseta* Pfingstl, 2015 (Supporting Information Figure S2c)

###### Present records

Thailand, Phang Nga province (Nang Thong Beach)

###### Biogeographic and morphological remarks

This species was originally described from the Maldives (Pfingstl, 2015b). The present specimens morphologically closely resemble to the Maldivian individuals. Their body size (451–469 μm) also overlaps with the original type specimens (446–465 μm).

### 3.4 Morphological description of new taxa

#### Selenoribatidae Schuster, 1963 *Indopacifica* Pfingstl, Shimano & Lienhard gen. nov. (Supporting Information Figures S3a,b)

##### Type species

*Indopacifica pantai* n. sp. Pfingstl, Shimano & Lienhard

##### Diagnosis

Strongly granular cerotegument. Lamellar ridges absent, only a pair of faint anteriorly converging cuticular elevations on interlamellar area. Sensillus clavate and distally spinose. Dorsosejugal suture incomplete. Fourteen pairs of notogastral setae, *c*_*2*_ absent. Inconspicuous light spot on anterior border of notogaster. Median cuticular deepening on epimeron I and inconspicuous semicircular deepening on epimeron III. Epimeral setation 1–0–1–1, three pairs of genital setae, aggenital setae absent. Lyrifissure *iad* oblique flanking anterior corner of anal opening. Three or two pairs of adanal setae, two pairs of anal setae. Coronal setae on ovipositor present. Legs monodactylous, femora with ventral carina. Famulus on tarsus I blunt, short and conical rod.

#### 3.4.1 *Indopacifica pantai* Pfingstl, Shimano & Lienhard n. sp

3.4.1.1

###### Diagnosis

Cerotegument granular with loosely distributed larger granules surrounded by smaller granules. Sensillus clavate, distally spinose. Notogaster rounded in dorsal view with 14 pairs of setiform notogastral setae. Conspicuous hourglass‐shaped median longitudinal depression on epimeron I. Epimeral setation 1–0 1–1. Three pairs of genital setae, aggenital setae absent. Three pairs of adanal setae and two pairs of anal setae. Claws on legs with one proximoventral tooth.

###### Differential diagnosis

This species can be distinguished from the second species of this genus by the presence of an hourglass‐shaped median longitudinal depression on epimeron I, the presence of three adanal setae, a notogastral cerotegument with loosely distributed larger granules surrounded by smaller granules and its larger body size.

##### Description of adult

###### Measurements

Females (*n* = 23), length: 319–356 μm (mean 338 μm), width: 200–219 μm (mean 210 μm); males (*n* = 28), length: 319–338 μm (mean 328 μm), width: 191–212 μm (mean 204 μm). Integument. Color brown. Cerotegument of prodorsum, ventral region and legs granular. Notogastral cerotegument conspicuously granular with large granules surrounded by smaller granules.

###### Prodorsum (Figure 6a)

**Figure 6:**
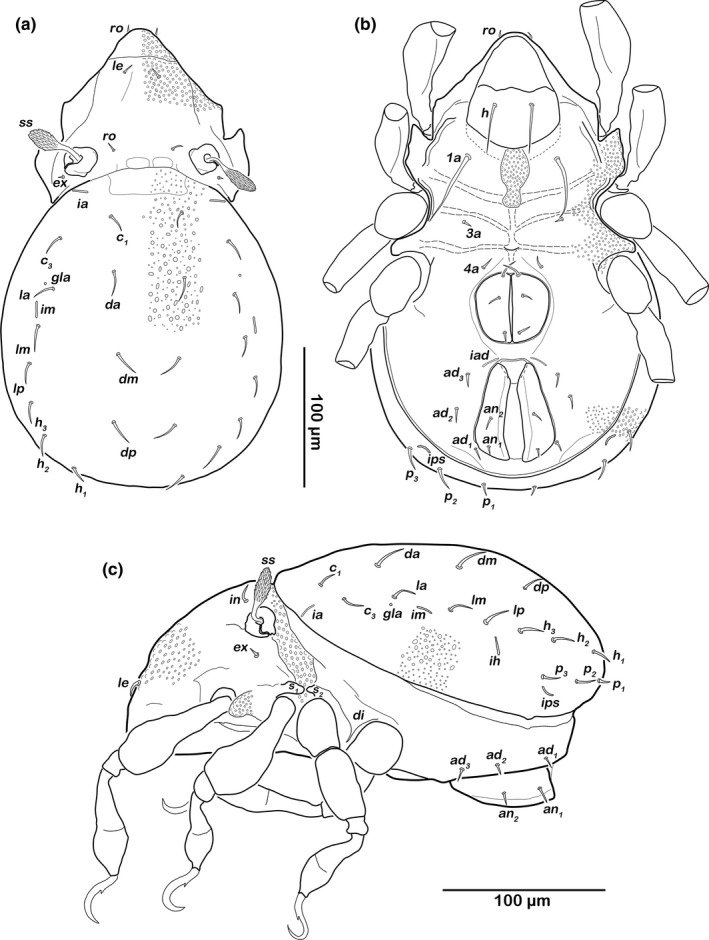
*Indopacifica pantai* n. sp. adult. (a) Dorsal view. (b) Ventral view, distal leg segments, and gnathosoma omitted. (c) Lateral view

Rostrum slightly rounded in dorsal view. Rostral (*ro*) and lamellar setae (*le*) simple and short. Interlamellar seta (*in*) thin, short, exobothridial seta (*ex*) very short. Bothridia large cups with lateral incision. Sensillus clavate, distally spinose.

###### Gnathosoma

Palp setal formula 0–2–1–3–9 (including solenidion). Palpfemur with paraxial porose area. Solenidion ω on tarsus not associated with eupathidium *acm*. Chelicera chelate, with interlocking teeth. Setae *cha* and *chb* of approximately same length, both dorsally slightly pectinate. Distal part of rutellum developed as thin triangular slightly curved inward membrane with longitudinal incision. Setae *a* and *m* long, smooth. Mentum regular, finely granular, seta *h* simple, long.

###### Notogastral region (Figures 6a,c)

Notogaster oval in dorsal view. Dorsosejugal suture incomplete. Fourteen pairs of thin, simple and short notogastral setae (length 12–16 μm), *c*_*1*_ *, c*_*3*_, *da*,* dm*,* dp*,* la*,* lm*,* lp*,* h*_*1–3*_, *p*_*1–3*_; *c*_*2*_ absent.

###### Lateral aspect (Figure 6c)

Pedotectum I present, round, small. Lateral enantiophysis consisting of two opposite rounded projections *S*_*1*_ and *S*_*2*_. Discidium *di* developed as prominent triangular bulge.

###### Podosoma and venter (Figure 6b)

Median longitudinal, hourglass‐shaped deepening on epimeron I, covered with fine granules. Epimeral setation 1–0–1–1. Three pairs of short, fine genital setae. Aggenital seta absent. Two pairs of short anal setae *an*_*1–2*_. Preanal organ triangular in ventral view, interior part anchor‐shaped. Three pairs of simple adanal setae *ad*_*1–3*_. Lyrifissure *iad* slightly oblique, next to anterior border of anal orifice.

###### Legs (Figure 7)

**Figure 7:**
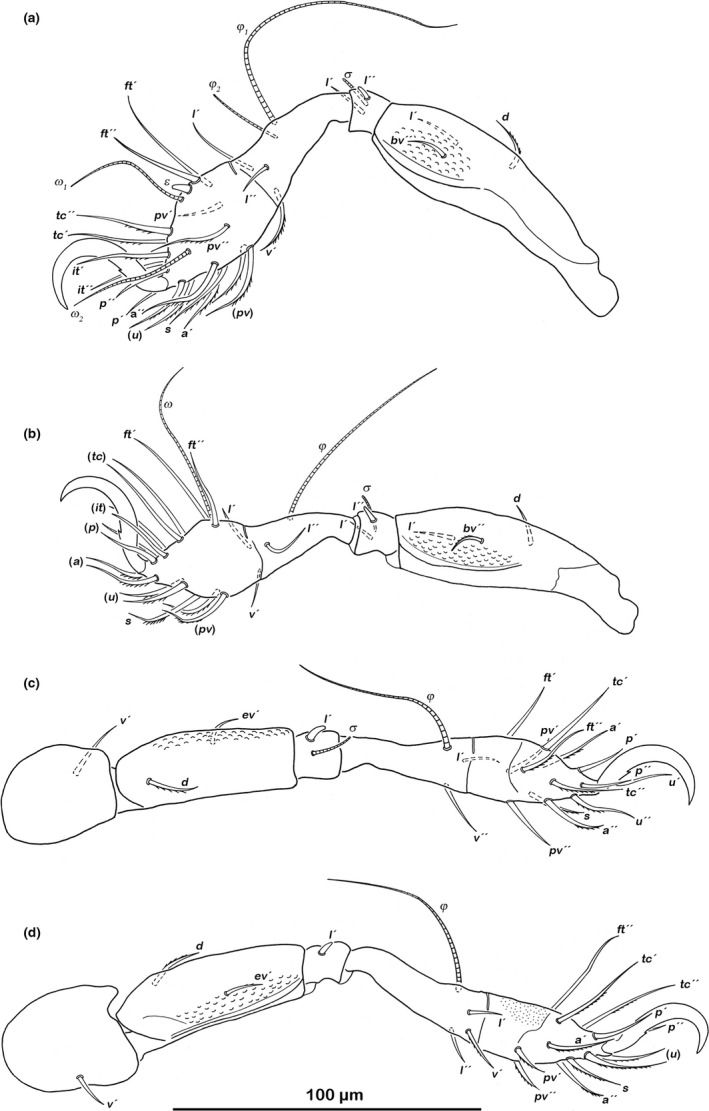
*Indopacifica pantai* n. sp. adult left legs, antiaxial view. (a) Leg I. (b) Leg II. (c) Leg III. (d) Leg IV

Monodactylous. Long hook‐like claws with one small proximoventral tooth. Cerotegument granular. Femora with ventral carinae. No porose areas detectable. Antiaxial lateral seta *l* on genua II and III slightly thickened and blunt. Setation and solenidia: Leg I (0–3–2–3–18) (1–2–2), leg II (0–3–2–3–16) (1–1–1), leg III (1–2–1–2–13) (1–1–0), leg IV (1–2–1–3–12) (0–1–0).

##### Type material

###### Holotype

Adult female, MY_17: Malaysia, Penang, Pantai Pasir Panjang, preserved in ethanol, deposited in the collection of the Naturhistorisches Museum Wien/NHM Vienna (NHMW 28672). Four paratypes (two males, two females) from the same sample and two paratypes (one male, one female) from Thailand, Phang Nga province, Nang Thong Beach deposited at the Princess Maha Chakri Sirindhorn Natural History Museum (PSU‐Museum) and additional specimens in the collections of the Institute of Biology, University of Graz.

##### Etymology

The generic name “*Indopacifica*” is given as noun and refers to the geographic area where this taxon is distributed. The specific epithet “*pantai*” is the Malay word for coast or beach and is given as noun in apposition.

##### Biogeographic and morphological remarks

Besides the present records in Thailand and Malaysia, this species also occurs in Singapore (Pulau Ubin), whereas these specimens were already mentioned as undetermined Selenoribatidae in Pfingstl (2015a). No geographic variations could be detected.

#### 3.4.2 *Indopacifica parva* Pfingstl, Shimano & Lienhard n. sp

3.4.2.1

###### Diagnosis

Cerotegument overall granular with densely packed larger granules surrounded by smaller granules. Sensillus clavate, distally spinose. Lamellar ridges absent, only a pair of faint anteriorly converging cuticular elevations on interlamellar area. Notogaster slightly oval in dorsal view with 14 pairs of setiform setae. Small median circular depression on epimeron I. Epimeral setation 1–0–1–1. Three pairs of genital setae, aggenital setae absent. Two pairs of anal‐ and adanal setae. Claws on legs with one proximoventral tooth.

###### Differential diagnosis

*Indopacifica parva* can be distinguished from *I. pantai* by the presence of a small median circular depression on epimeron I, the absence of a third pair of adanal seta, a notogastral cerotegument with densely packed larger granules surrounded by smaller granules, and its obvious smaller body size (on average 50 μm smaller).

##### Description of adult

###### Measurements

Females (n = 6), length: 268–312 μm (mean 290 μm), width: 163–192 μm (mean 176 μm); male (n = 2), length: 259–296 μm (mean 278 μm), width: 160–175 μm (mean 168 μm).

###### Integument

Color brown. Cerotegument of all body parts with densely distributed large granules, notogastral cerotegument consisting of larger granules surrounded by smaller granules.

###### Prodorsum (Figure 8a)

**Figure 8:**
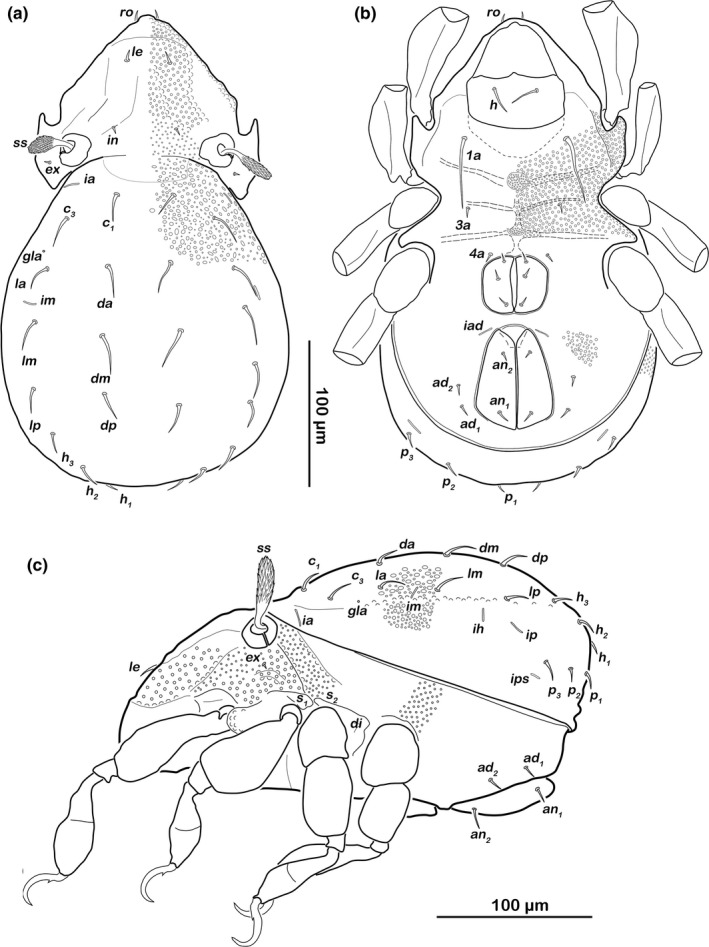
*Indopacifica parva* n. sp. adult. (a) Dorsal view. (b) Ventral view, distal leg segments, and gnathosoma omitted. (c) Lateral view

Rostrum rounded in dorsal view, slightly projecting anteroventrally in lateral view. Rostral (*ro*) and lamellar setae (*le*) simple and short. Interlamellar seta (*in*) thin, short, exobothridial seta (*ex*) minute. Bothridia large cups with lateral incision. Sensillus clavate, distally spinose.

###### Gnathosoma

As in *I. pantai*.

###### Notogastral region (Figures 8a,c)

Notogaster pear shaped in dorsal view. Dorsosejugal suture incomplete. Fourteen pairs of thin, simple notogastral setae (length 10–13 μm), *c*_*1*_ *, c*_*3*_, *da*,* dm*,* dp*,* la*,* lm*,* lp*,* h*_*1–3*_, *p*_*1–3*_; *c*_*2*_ absent. Orifice of opisthonotal gland *gla* laterally and between seta *c*_*3*_ and *la*, surrounded by a cuticular bulge.

###### Lateral aspect (Figure 8c)

Cerotegument granular, larger granules on pedotectum I, discidium *di* and in acetabular regions. Pedotectum I present, round, small. Lateral enantiophysis consisting of two opposite rounded projections *S*_*1*_ and *S*_*2*_. Discidium *di* developed as prominent triangular bulge.

###### Podosoma and venter (Figure 8b)

Small median circular depression on epimeron I and inconspicuous semicircular deepening on epimeron III. Epimeral setation 1–0–1–1. Three pairs of short, fine genital setae. Aggenital seta absent. Two pairs of short anal setae *an*_*1–2*_. Preanal organ triangular in ventral view, interior part anchor‐shaped. Two pairs of simple adanal setae *ad*_*1–2*_. Lyrifissure *iad* oblique, next to anterior border of anal orifice.

###### Legs (Figure 9)

**Figure 9:**
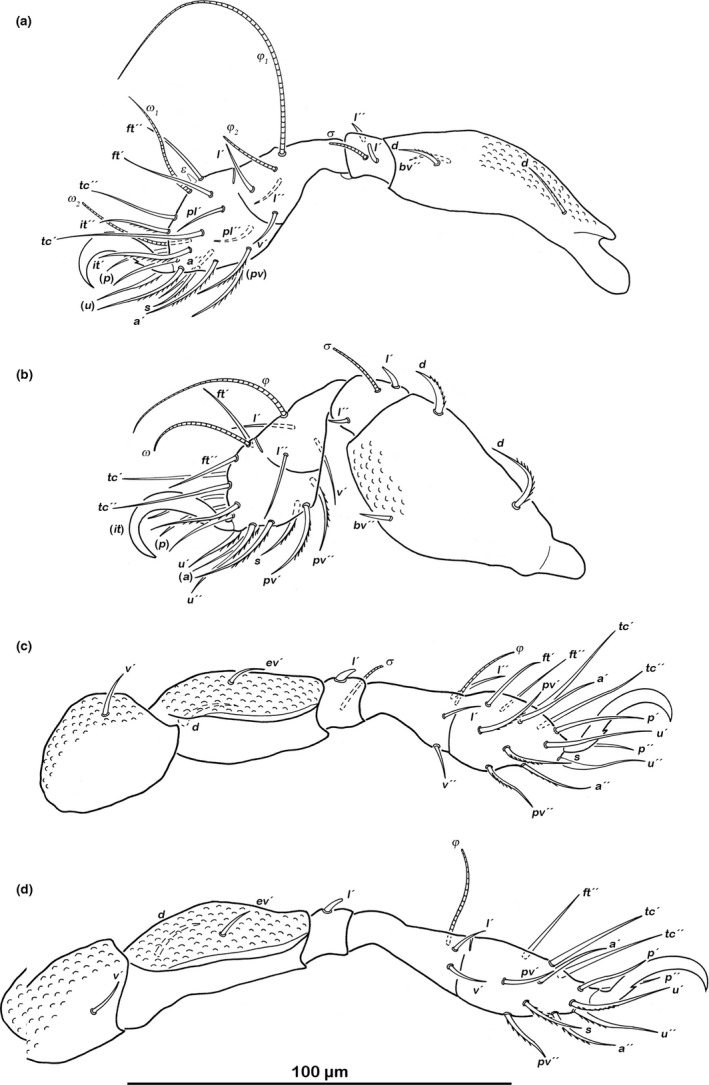
*Indopacifica parva* n. sp. adult left legs, antiaxial view. (a) Leg I. (b) Leg II. (c) Leg III. (d) Leg IV

Monodactylous. Long hook‐like claws with one small proximoventral tooth. Femora with ventral carinae. No porose areas detectable. Setation and solenidia: Leg I (0–3–2–3–18) (1–2–2), leg II (0–3–2–3–16) (1–1–1), leg III (1–2–1–3–13) (1–1–0), leg IV (1–2–1–2–12) (0–1–0).

##### Type material

###### Holotype

Adult female; TH_09: Thailand, Phang Nga province, Nang Thong Beach; preserved in ethanol, deposited in the collection of the Naturhistorisches Museum Wien/NHM Vienna (NHMW 28673). Three paratypes from the same sample deposited at the Princess Maha Chakri Sirindhorn Natural History Museum (PSU‐Museum); additional specimens in the collections of the Institute of Biology, University of Graz.

##### Etymology

The specific epithet is the Latin word “*parva*” meaning smaller and refers to the relatively small body size of this species.

##### Biogeographic and morphological remarks

In this study, this species was found at a single location in Thailand and on the Island of Langkawi. Other specimens were also confirmed to be present at the coasts of Singapore (Pulau Ubin) (Pfingstl unpublished). The specimens from Singapore are more or less identical but their anal orifice is framed by small cuticular furrows, a character completely lacking in the individuals from Thailand.

## 4 DISCUSSION

### 4.1 Diversity

The present study revealed the presence of six intertidal oribatid mite species from three different genera, *Alismobates*,* Fortuynia,* and *Indopacifica*, belonging to two different families, the Fortuyniidae and Selenoribatidae, in the Andaman and Strait of Malacca coastal region. The fortuyniid species *F. smiti*,* F. longiseta,* and *A. pseudoreticulatus* were already known to science but have not been recorded from this specific area before. The selenoribatid *I. pantai* n. sp. and *I. parva* n. sp., on the other hand, are newly discovered species that even represent a separate genus. A third *Fortuynia* species was also detected by molecular genetic means (see Figure 2) but we refrain from naming and describing this species because we did not have enough specimens for a thorough morphological analysis. Nonetheless, a preliminary morphological assessment of body parts remaining after DNA extraction suggests that this species is very similar to *F. smiti*.

**Figure 10:**
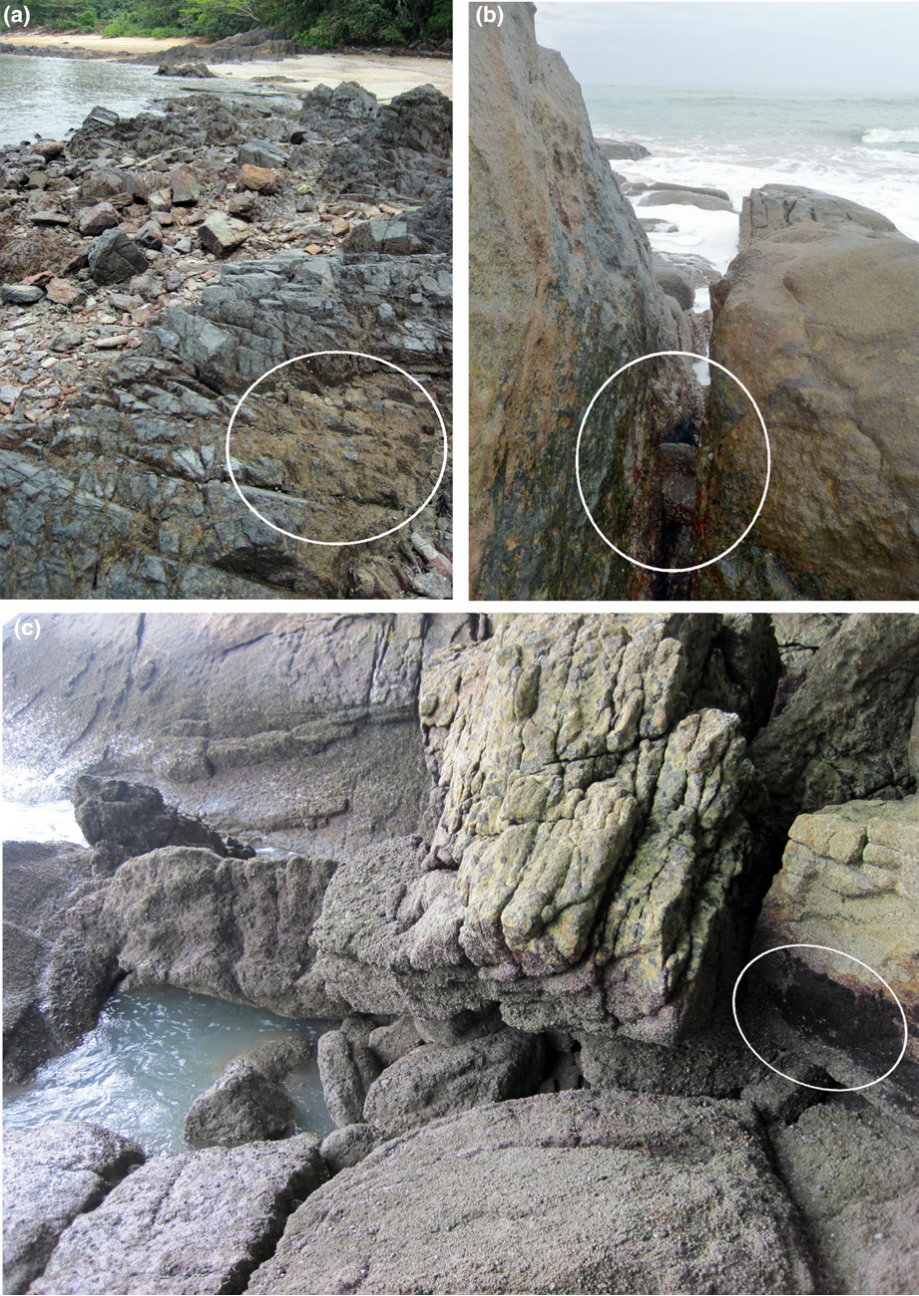
Photographs of sample locations highlighting the littoral microhabitats occupied by the mites; white ellipses indicate the alga mats that were sampled. (a) Langkawi, Datai Bay (MY_12), rocks, and boulder covered with intertidal algae (best seen in the right lower corner). (b) Phang Nga province, Nang Thong Beach (TH_09), crevice overgrown with algae. (c) Penang, Pantai Pasir Panjang (MY_17), rocky shore covered with barnacles and algae

The found high diversity demonstrates that oribatid mites are a common part of the Andaman intertidal fauna, and the occurrence of at least two species in the majority of the samples (Supporting Information Table S3) shows that the superficially meager littoral habitat (Figure 10a,b,c) offers enough ecological niches to support various taxa at the same time. Given the low number of locally restricted sampling sites of this study, it is assumable that further species may be present along the coasts of this geographic area, especially mangrove forests, which were more or less completely neglected in this study, may harbor further taxa.

### 4.2 Population structure

**Figure 11:**
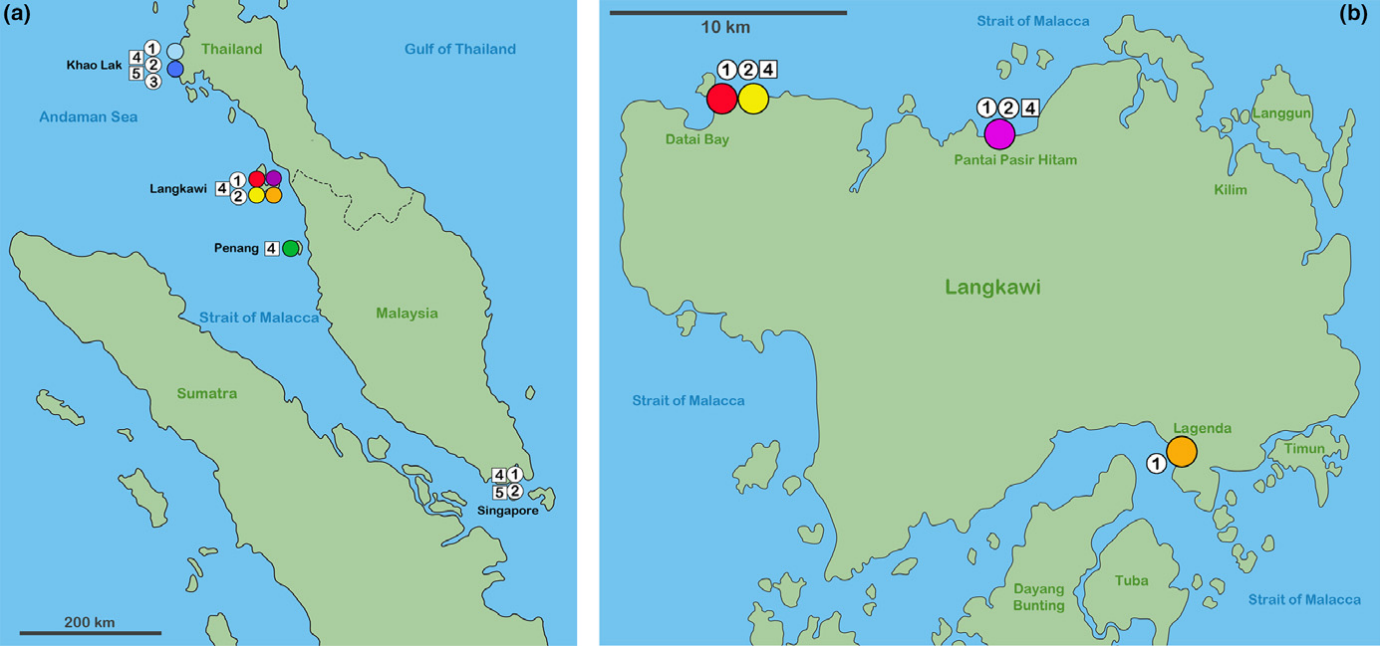
Distribution maps showing records of intertidal mite taxa. (a) In the Andaman Sea and the Strait of Malacca (records in Singapore are taken from Pfingstl, 2015b and from unpublished data). (b) On the island of Langkawi (Malaysia). Circles represent fortuyniid taxa; squares indicate selenoribatid taxa; numbers refer to species: 1—*Alismobates pseudoreticulatus*, 2—*Fortuynia smiti*, 3—*Fortuynia longiseta*, 4—*Indopacifica pantai* n. sp., 5—*Indopacifica parva* n. sp.; colors are the same as used in haplotype networks and refer to sample locations

The *A. pseudoreticulatus* populations from Langkawi (Figure 11b) show slight morphological divergences in body length and posterior body width. Size differences are usually supposed to be nongenetic intraspecific variation caused by ecological factors (Jungers, Falsetti, & Wall, 1995), and the same may be true for the present populations. The population with larger specimens was found on a natural rock formation with extensive mats of algae, whereas the population with the smaller specimens originated from artificial concrete structures overgrown with small patches of algae. The latter habitat could only have been recently colonized by the algae and the mites, and therefore, ecological conditions may be less favorable there than in the established natural habitat. However, after size correction, there is still a slight shape variation to be found between the populations, which may be an indication of genetic diversification. *COI* sequence data show a more or less clear geographic pattern, suggesting that there is not much genetic exchange between the populations of this single small island. Nevertheless, one of the Langkawi haplotypes was also found at the coast of Thailand, which indicates that there has been at least one recent long distance transport.

This is in contrast to *I. pantai* n. sp. where various haplotypes can be found without a strict geographic pattern. Closer haplotypes are present at far distant locations which points to gene flow among the populations from Penang, Langkawi, and Thailand. Despite this gene flow, morphometric data indicate slight size and shape variation between the far distant populations from Thailand and Penang. Differences in habitat, as shown in *A. pseudoreticulatus*, could not be detected; therefore, genetic drift may be primarily responsible for the variation.

### 4.3 Distribution patterns and dispersal

All found species show wide distribution areas in the Andaman region and the Strait of Malacca, some of them even stretching beyond the borders of this geographic region. The distribution of *A. pseudoreticulatus*,* F. smiti,* and the two *Indopacifica* species reaches from the western coast of Thailand downwards to the southernmost tip of the Malaysian peninsula (Figure 11a). *Fortuynia smiti* was originally found in New Caledonia (Ermilov et al., 2013) and hence may be plausibly occurring in the whole Indomalayan realm. *Fortuynia longiseta* was presently only detected on the coast of Thailand but is also known to occur in the Maldives (Pfingstl, 2015b) and therefore may be widely distributed in the Indian Ocean.

Despite their minute size and wingless body, these arthropod species seem to be good dispersers that are able to cross oceanic barriers and colonize vast shorelines. Hydrochorous dispersal, that is, drifting along ocean currents, is supposed to be the most important way of long distance transport in these small taxa (Coulson et al., 2002; Pfingstl, 2013b; Schatz, 1991). Sea surface currents in the Andaman Sea and Strait of Malacca change their directions according to the tropical monsoon seasons (Kiran, 2017; Wyrtki, 1961), and this periodic reversal may allow multidirectional gene flow between populations. This could explain why single species show wide‐ranging distributions with no noteworthy distinct morphological variation between geographic areas.

Unfortunately, genetic data for *Fortuynia* species were only available for single populations of each species; therefore, haplotype network data tell us nothing about dispersal or gene flow between different populations of a species, but molecular genetic data of *I. pantai* n. sp. indicate recent gene flow between populations from Thailand, Langkawi, and Penang and confirm this species as good disperser. *Alismobates pseudoreticulatus*, on the other hand, may have weaker dispersal abilities as indicated by the clear geographic pattern of populations from the Island of Langkawi, but recent long distance dispersal for this species is also confirmed by the presence of the same haplotype in Langkawi and in Thailand. The reasons why *I. pantai* shows obviously better dispersal abilities than *A. pseudoreticulatus* are presently unknown. Anyway, a slight trend to morphological diversification can be observed in all morphometrically studied populations of *I. pantai* n. sp. and *A. pseudoreticulatus*, which may be an indication of genetic drift. Therefore, the frequency of successful dispersal events may be low and strongly depend on local sea current conditions and dispersal abilities of the respective species. Similar morphological differentiations were observed in intertidal oribatid mites from the Galapagos and the Hawaii archipelago and were suggested to be the results of restricted gene flow between the island populations (Pfingstl & Baumann, 2017; Pfingstl & Jagersbacher‐Baumann, 2016).

### 4.4 Systematics

The new genus *Indopacifica* can be clearly assigned to the Selenoribatidae based on the epimeral setal formula 1–0–1–1 and the partial fusion of tibia and tarsus, characters unique to this family (Grandjean, 1966). In the following paragraph, the new taxon will be compared to each selenoribatid genus to justify the erection of this new genus on a morphological basis (for a tabular overview see Table 4). *Indopacifica* differs from the selenoribatid genus *Arotrobates* by the incomplete dorsosejugal suture (vs. complete), the absence of epimeral carinae (vs. presence), the presence of epimeral foveae (vs. absence), and the anterior position and oblique orientation of lyrifissure *iad* (vs. posterior and longitudinal). The new genus can be distinguished from *Carinozetes* by the incomplete dorsosejugal suture (vs. complete), the absence of ventral carinae (vs. presence), 14 pairs of notogastral setae (vs. 15), and the oblique orientation of *iad* (vs. transversal). *Indopacifica* differs from *Psednobates* in lacking lamellar ridges (vs. presence), the presence of epimeral foveae (vs. absence), and the possession of anal setae (vs. complete absence); it is distinct from *Rhizophobates* by the oblique orientation of *iad* (vs. longitudinal) and the presence of epimeral foveae (vs. absence). The latter character state and the possession of two anal setae instead of one, the presence of *k*‐setae on the ovipositor (vs. absence), and the knob‐like famulus (vs. rod‐like) clearly separate the new genus from *Schusteria*. The genus *Selenoribates* shows lamellar ridges, notogastral depressions, and a complete dorsosejugal suture, all traits lack or differ in *Indopacifica*. *Thalassozetes* also shows lamellar ridges and a complete dorsosejugal suture and therefore also differs considerably from the new genus. Finally, *Indopacifica* can be distinguished from *Thasecazetes* by the absence of lamellar ridges (vs. presence), 14 pairs of notogastral setae (vs. 15), and the oblique orientation of *iad* (vs. transversal).

**Table 4:** Comparison of diagnostic morphological features of all selenoribatid genera

	*Arotrobates*	*Carinozetes*	*Psednobates*	*Indopacifica* nov. gen.	*Rhizophobates*	*Schusteria*	*Selenoribates*	*Thalassozetes*	*Thasecazetes*
Lamellar ridges	Absent	Absent	**Present**	**Absent**	Absent	Absent	**Present**	**Present**	**Present**
Dorsosejugal suture	Complete	Complete	Complete	**Incomplete**	Incomplete	Incomplete	Complete	Complete	**Incomplete**
Notogastral setae	14–15	**15**	14	14	14	**15**	14	**13**–**14**	15
Notogastral depressions	Absent	Variable	**Absent**	Absent	**Absent**	Absent	**Present**	Variable	Absent
Epimeral carinae	**Present**	**Present**	Absent	Absent	**Absent**	**Absent**	Absent	Absent	Absent
Epimeral foveae	Absent	Present	Absent	Present	**Absent**	Absent	**Present**	Variable	Present
Anal setae	1–2	2	0	2	2	**1**	2–3	2	1
*Iad* position relative to AN	**Posterior**	Anterior	Anterior	Anterior	Anterior	Anterior	Anterior	Anterior	Anterior
*iad* orientation	Longitudinal	**Transversal**	**Oblique**	**Oblique**	**Longitudinal**	Variable	**Oblique**	Variable	**Transversal**
k‐setae on ovipositor	?	Present	?	**Present**	?	**Absent**	Present	Present	?

*Notes.* Characters in bold represent traits or trait combinations unique to the respective genus.

?: no information available.

Despite the mosaic and sometimes overlapping distribution of diagnostic traits, there are enough characters clearly separating *Indopacifica* from all the other selenoribatid genera. Assessing generic relationships within the Selenoribatidae based on morphological data is very difficult due to the above‐mentioned mosaic traits but *Indopacifica* most likely is closely related to *Rhizophobates* and *Schusteria* because the absence of lamellar ridges in combination with an incomplete dorsosejugal suture are characters shared only by these three taxa.

Based on molecular genetic data, *Indopacifica* forms a well‐supported single clade, and hence, *18S* ribosomal RNA sequences clearly confirm the distinctness of the new genus and place it close to *Thalassozetes shimojanai* (Karasawa & Aoki, 2005). The latter was originally described as *Rhizophobates shimojanai* (Karasawa & Aoki, 2005) but was later transferred to *Thalassozetes* Schuster, 1963 without any justification (Subías, 2004). Unfortunately, Iseki and Karasawa (2014) adopted this invalid classification and the available DNA sequence was saved in GenBank under the wrong name. So in actual fact, *Indopacifica* is closest related to *Rhizophobates* and not to *Thalassozetes*.

Unfortunately, genetic data of a real *Thalassozetes* are lacking so far, the same is true for the remaining selenoribatid genera, *Arotrobates*,* Carinozetes*,* Psednobates,* and *Selenoribates;* therefore, reliable phylogenetic assessments are not feasible at present.

## APPENDIX 1

### GenBank accession numbers for *COI, EF‐1* α *,* and *18S* sequences comprising all specimens included in genetic investigations

**Table:**

Species	Sample ID	GenBank accession no.		
		*COI*	*EF‐1*α	*18S*
*Alismobates pseudoreticulatus*	A_MY_05_1	MH285595		
	A_MY_05_10	MH285596		
	A_MY_05_3	MH285597	MH285676	
	A_MY_05_4	MH285598		
	A_MY_05_5	MH285599		
	A_MY_05_6	MH285600		
	A_MY_05_7	MH285601		
	A_MY_05_8	MH285602		
	A_MY_05_9	MH285603		
	A_MY_07_1	MH285604		
	A_MY_07_10	MH285605		
	A_MY_07_12	MH285606		
	A_MY_07_13	MH285607		
	A_MY_07_2	MH285608		
	A_MY_07_3	MH285609	MH285677	
	A_MY_07_4	MH285610	MH285678	
	A_MY_07_5	MH285611		
	A_MY_07_6	MH285612		
	A_MY_07_7	MH285613	MH285679	MH285696
	A_MY_07_8	MH285614		
	A_MY_07_9	MH285615		
	A_MY_11_2	MH285616		
	A_MY_11_4	MH285617		
	A_MY_11_5	MH285618		
	A_TH_06_1	MH285619		
*Fortuynia* sp.	F_MY_07_2	MH285621		
	F_MY_07_2a	MH285622		
	F_MY_07_3	MH285623		
	F_MY_07_4	MH285624		
	F_MY_07_6	MH285625		
	F_MY_07_7	MH285626	MH285674	
	F_MY_07_8N	MH285627	MH285675	MH285695
*Fortuynia smiti*	F_A_MY_11_3	MH285620	MH285680	
	F_MY_11_1N	MH285628	MH285681	MH285694
	F_MY_11_2	MH285629		
	F_MY_12_1	MH285630		
	F_MY_12_2	MH285631		
	F_MY_12_3N	MH285632		
	F_S_MY_12_1	MH285641		
	F_S_MY_12_1a	MH285642		
	F_S_MY_12_1b	MH285643		
	F_TH_06_1N	MH285633		
*Fortuynia longiseta*	F_TH_06_2	MH285634		
	F_TH_06_4	MH285635	MH285682	
	F_TH_06_5	MH285636	MH285683	MH285693
*Indopacifica pantai* sp. nov.	S_MY_07_1	MH285637		
	S_MY_07_2	MH285638		
	S_MY_07_4	MH285640		
	S_MY_17_10	MH285644		
	S_MY_17_11	MH285645		
	S_MY_17_12	MH285646		
	S_MY_17_13	MH285647		
	S_MY_17_2	MH285648		
	S_MY_17_3	MH285649		
	S_MY_17_4	MH285650	MH285684	
	S_MY_17_5	MH285651		
	S_MY_17_6	MH285652	MH285685	MH285692
	S_MY_17_7	MH285653		
	S_MY_17_8	MH285654	MH285686	MH285691
	S_MY_17_9	MH285655		
	S_MY_17_9a	MH285656		
	S_TH_06_1	MH285657		
	S_TH_09_1	MH285658		
	S_TH_09_10	MH285659		
	S_TH_09_11	MH285660		
	S_TH_09_12	MH285661		
	S_TH_09_2	MH285662		
	S_TH_09_3	MH285663		
	S_TH_09_4	MH285664		
	S_TH_09_5	MH285665	MH285687	
	S_TH_09_6	MH285666		
	S_TH_09_7	MH285667		
	S_TH_09_8	MH285668		
	S_TH_09_8a	MH285669		
	S_TH_09_9	MH285670		
*Indopacifica parva* sp. nov.	S2_MY_07_3	MH285639		
	S2_TH_09_1	MH285671	MH285689	MH285690
	S2_TH_09_2	MH285672		
	S2_TH_09_3	MH285673	MH285688	
*Alismobates reticulatus*				AB818526.1 a
*Aquanothrus sp*.				KX397627.1 b
*Litoribates bonairensis* (1)				MF997503^c^
*Litoribates bonairensis* (2)				MF997502^c^
*Thasecazetes falcidactylus*				MF997501^c^
*Schusteria littorea*				HM070345.1 d
*Limnozetes rugosus*				KX397636 b
*Hydrozetes thienemanni*				KX397633 b
*Scapheremaeus palustris*				EU433989 e
*Fortuynia rotunda*				AB818525.1 a
*Thalassozetes shimojanai*				AB818524.1 a

*Notes*

Sequences generated in this study appear in bold. ^a^Iseki and Karasawa (2014). ^b^Krause et al. (2016). ^c^Pfingstl et al. (2017). ^d^Pepato, da Rocha, and Dunlop (2010). ^e^Schaefer, Norton, Scheu, and Maraun (2010).
