# Supplementary material for: Systematics, genetics, and biogeography of intertidal mites (Acari, Oribatida) from the Andaman Sea and Strait of Malacca
Source: J Zool Syst Evol Res. 2018 Sep 4;57(1):91–112. doi: 10.1111/jzs.12244 (PMC6378605; doi:10.1111/jzs.12244)
Supplement: Supplementary file 1 [file JZS-57-91-s001.pdf]

Supporting information

Systematics, genetics and biogeography of intertidal mites (Acari, Oribatida) from the Andaman Sea and Strait of Malacca

Tobias PFINGSTL, Andrea LIENHARD, Satoshi SHIMANO, Zulfigar Bin YASIN, Aileen Tan SHAU-HWAI, Sopark JANTARIT, Booppa PETCHARAD

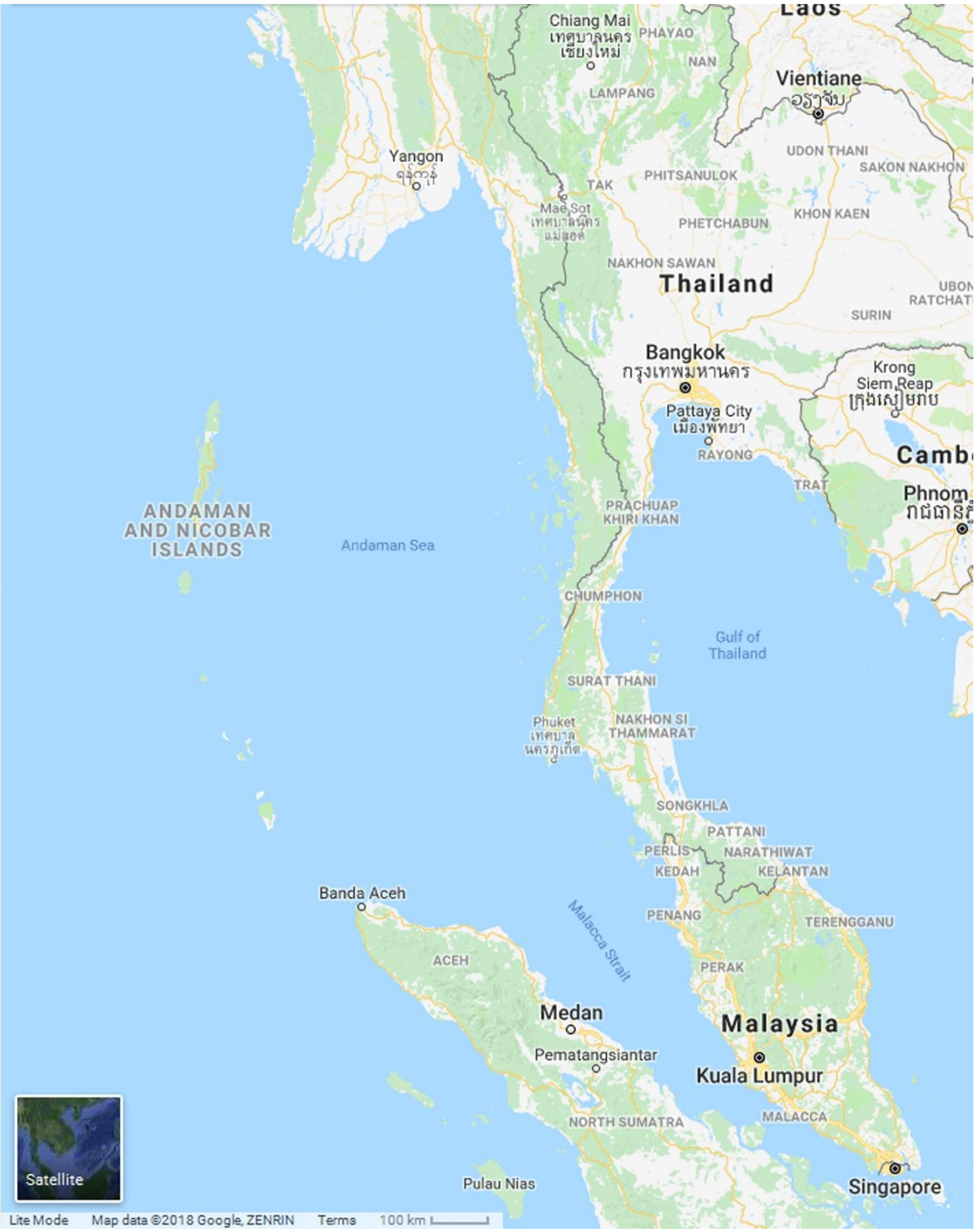

FIGURE S1 Overview map (copyright google maps) of the Andaman Sea and adjacent regions.

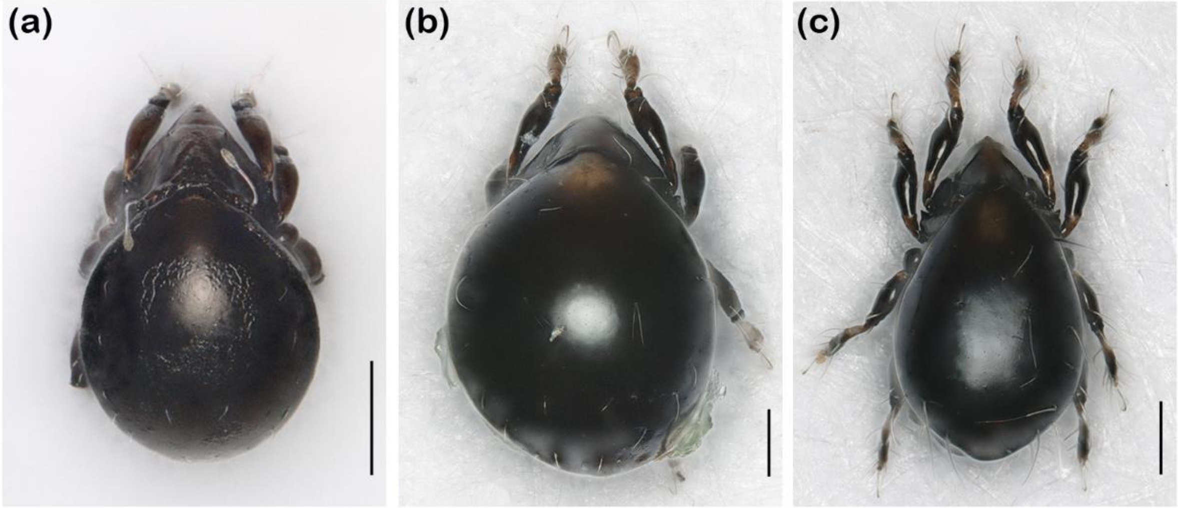

FIGURE S2 Photographs of found fortuyniid species in dorsal view (stacked stereomicroscopic images); scale bars 100µm. (a) *Alismobates pseudoreticulatus*. (b) *Fortuynia smiti*. (c) *Fortuynia longiseta*.

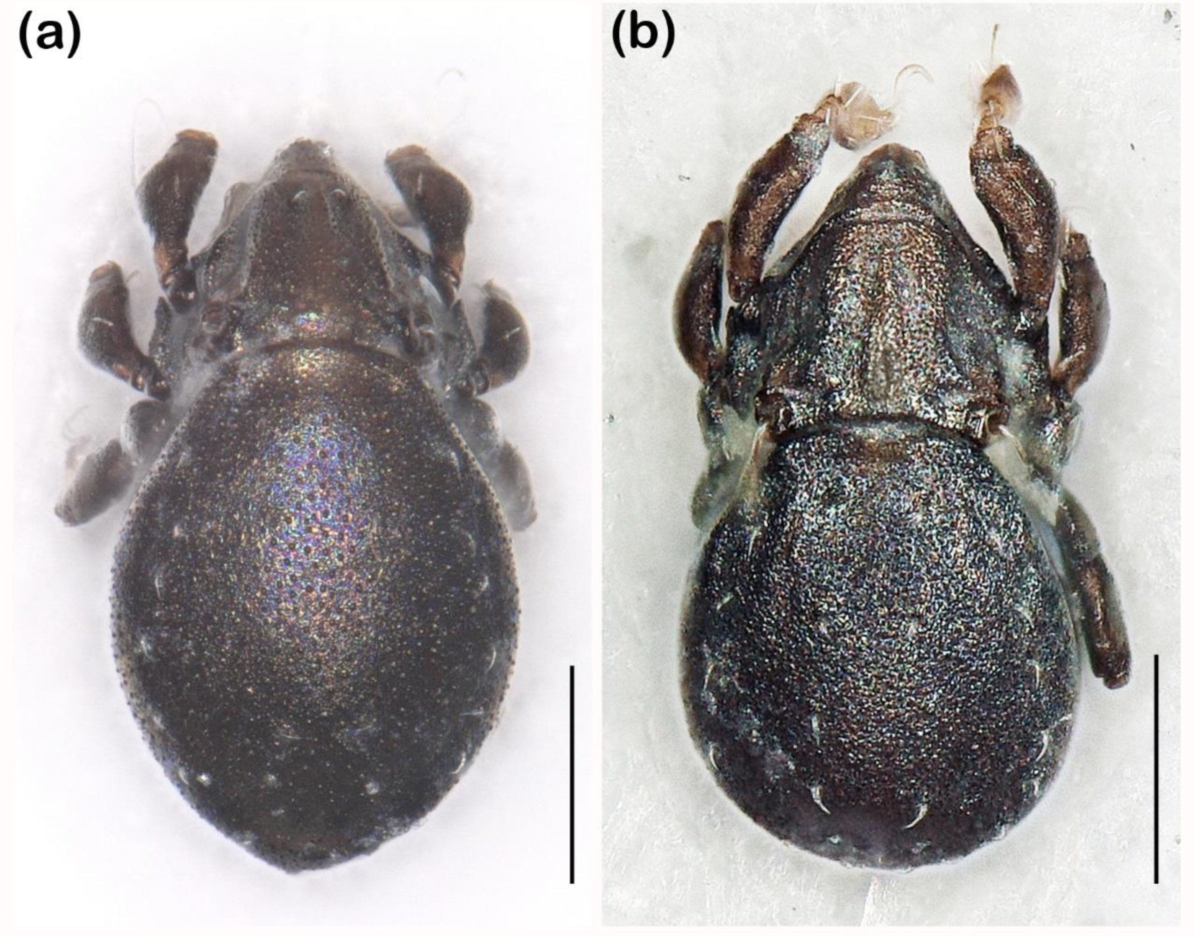

FIGURE S3 Photographs of new selenoribatid species in dorsal view (stacked stereomicroscopic images); scale bars 100µm. (a) *Indopacifica pantai* sp. nov. (b) *Indopacifica parva* sp. nov.

Supporting Table S1. Loadings of the PCA for the three *Alismobates pseudoreticulatus* populations from Langkawi. Values higher than 0.5 are given in bold.

	raw data			size corrected		
	PC 1	PC 2	PC 3	PC 1	PC 2	PC 3
bl	0.15	0.21	-0.02	0.28	**0.63**	**0.58**
dPtI	0.13	0.02	0.14	-0.04	0.01	0.20
db	0.17	0.12	0.18	0.00	-0.05	-0.12
ll	**0.62**	**-0.56**	-0.03	-0.33	-0.03	0.04
nw_{da}	0.28	0.16	0.07	-0.04	**0.63**	-0.55
nw_{dm}	0.17	0.26	0.04	0.27	0.26	0.03
nw_{dp}	-0.08	**0.58**	0.08	**0.85**	-0.28	-0.22
cl	0.02	-0.01	0.48	0.03	-0.13	0.33
cw	0.19	-0.03	0.15	-0.07	-0.10	0.01
dcg	0.17	0.14	-0.73	0.02	-0.06	0.15
dac3	0.10	0.04	-0.04	-0.01	-0.13	0.26
gl	0.39	0.25	0.31	-0.01	0.02	-0.19
gw	0.26	0.24	-0.09	0.01	0.06	-0.02
al	0.23	0.21	-0.20	0.03	0.06	-0.08
aw	0.27	0.08	-0.04	-0.05	-0.06	-0.10

Supporting Table S2. Loadings of the PCA for the two *Indopacifica pantai* sp. nov. populations from Phang Nga (Thailand) and Penang (Malaysia). Values higher than 0.5 are given in bold.

	raw data			size corrected		
	PC 1	PC 2	PC 3	PC 1	PC 2	PC 3
bl	0.13	0.03	0.06	0.37	**0.62**	-0.37
dPtI	0.09	-0.02	0.13	0.21	0.09	0.25
db	0.12	0.00	0.53	0.09	-0.06	0.10
nw_{da}	0.15	0.04	0.07	-0.05	0.02	**0.78**
nw_{dm}	0.13	0.07	0.15	0.30	-0.18	-0.01
nw_{dp}	0.09	0.08	0.32	**0.60**	**-0.54**	-0.15
cl	0.05	0.08	-0.41	-0.04	0.40	0.00
cw	0.04	-0.01	0.08	0.11	0.11	0.05
efw	**0.61**	**-0.78**	-0.06	-0.14	0.03	0.01
dcg	-0.06	-0.02	**0.57**	0.38	0.03	0.14
dac3	0.08	0.01	0.16	0.18	0.08	0.16
gl	0.50	0.39	-0.07	-0.27	-0.20	-0.18
gw	0.50	0.44	-0.10	-0.27	-0.20	-0.28
al	0.07	0.04	-0.07	0.10	0.13	-0.05
aw	0.16	0.17	0.16	-0.05	-0.02	0.01

Supporting Table S3. Syntopic occurences of taxa found in the present study.

		A. pseudoreticulatus	*F. longiseta*	*F. smiti*	*I. pantai* sp. n.	*I. parva* sp. n.
Langkawi	Legenda (MY_05)	+				
	Hitam Pasir (MY_07)	+		+	+	
	Datai Bay (MY_11)	+		+		
	Datai Bay (MY_12)			+	+	
Penang	Pangjang Pasir (MY_17)				+	
Phang Nga	Nang Thong (TH_06)	+	+	+	+	
	Nang Thong (TH_09)				+	+

Supporting Table S4. Primers used in the present study.

	Primer	Sequence (5´-3´)	Source
COI			
	Mite COI-2F	TTY GAY CCI DYI GGR GGA GGA GAT CC	Otto & Wilson (2001)
	Mite COI-2R	GGR TAR TCW GAR TAW CGT NCG WGG TAT	Otto & Wilson (2001)
EF-1α			
	40.71F	TCN TTY AAR TAY GCN TGG GGT	Regier & Shultz (1997)
	52.RC	CCD ATY TTR TAN ACR TCY TG	Regier & Shultz (1997)
18S			
	18Sfw	CTTGTCTCAAAGATTAAGCCATGCA	Dabert et al. (2010)
	rev960	GACGGTCCAAGAATTTCAC	Dabert et al. (2010)
	fw770	ACTTTGAAAAAATTAGAGTGC	Dabert et al. (2010)
	rev18S	TGATCCTTCCGCAGGTTCACCT	Dabert et al. (2010)
	fw390*	AATCAGGGTTCGATTCCGGAGA	Dabert et al. (2010)
	rev480*	GTTATTTTTCGTCACTACCT	Dabert et al. (2010)
	fw1230*	TGAAACTTAAAGGAATTGACG	Skoracka & Dabert (2010)

*Primers only used for sequencing.
