# Supplementary material for: Systematics, genetics, and biogeography of intertidal mites (Acari, Oribatida) from the Andaman Sea and Strait of Malacca
Source: J Zool Syst Evol Res. 2018 Sep 4;57(1):91–112. doi: 10.1111/jzs.12244 (PMC6378605; doi:10.1111/jzs.12244)
Supplement: Supplementary file 2 [file JZS-57-91-s002.docx]

**Systematics, genetics and biogeography of intertidal arthropods (Acari, Oribatida) from the Andaman Sea and the Strait of Malacca**

Tobias Pfingstl, Andrea Lienhard, Satoshi Shimano, Zulfigar Bin Yasin, Aileen Tan Shau Hwai

This study revealed a relatively high diversity of intertidal oribatid mites in the Andaman Sea and Strait of Malacca, including the presence of the new genus *Indopacifica*. All species show wide distribution areas and molecular genetic data confirm recent gene flow among far distant populations indicating good dispersal abilities. Morphometric data show slight size and shape variations among different populations, which are suggested to be results of genetic drift and ecological differences within the habitat.

**
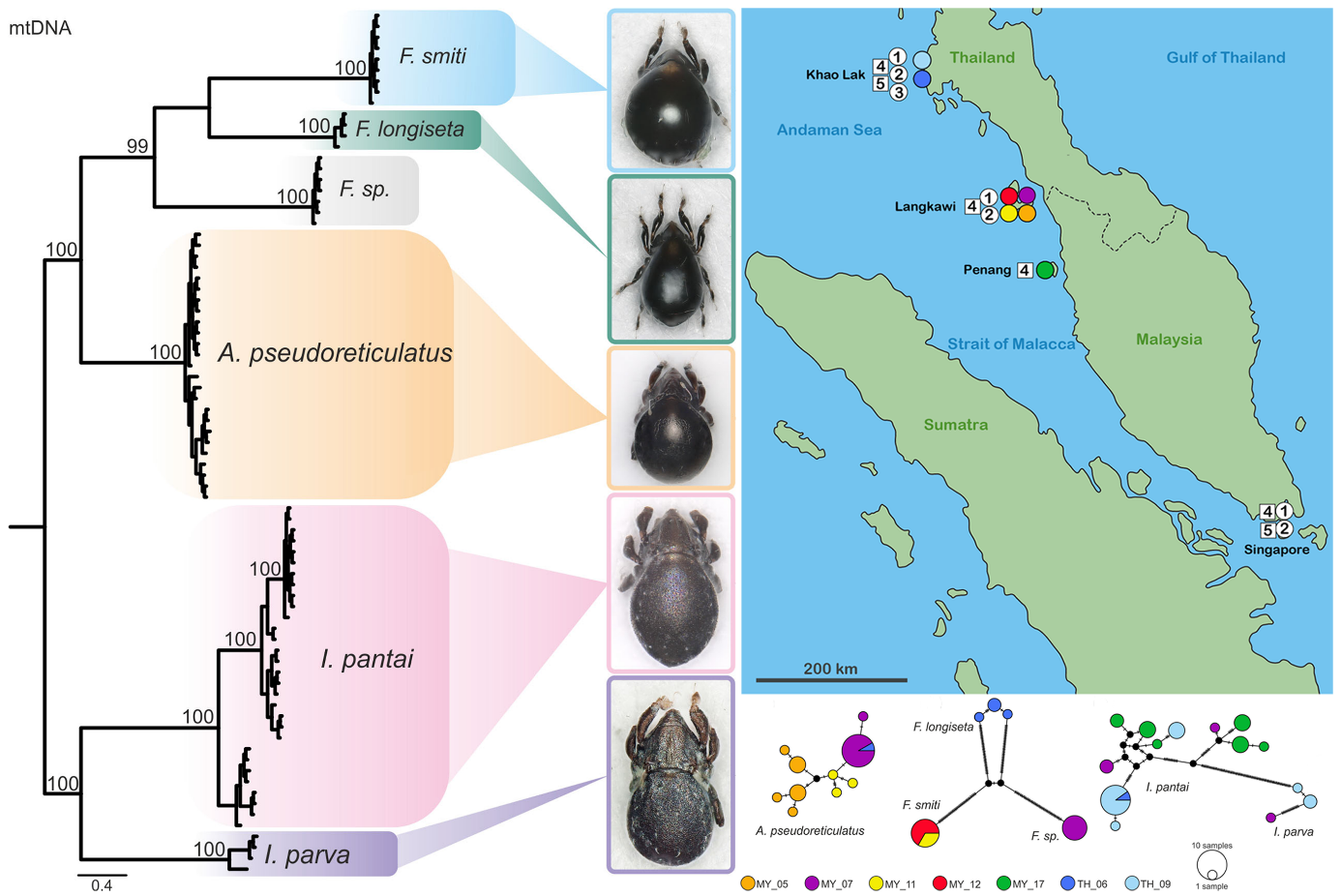
**
